# Social Media Use During Pregnancy: Risks and Benefits for Women’s Health

**DOI:** 10.7759/cureus.90410

**Published:** 2025-08-18

**Authors:** Nida Aftab

**Affiliations:** 1 Obstetrics and Gynaecology, Hywel Dda University Health Board, NHS Wales, Camarthen, GBR

**Keywords:** among pregnant women, baby care, body dissatisfaction, mother-baby relationship, pregnancy, prenatal care awareness, public health problems, risks and benefits, social media communication, women’s health

## Abstract

Social media use during pregnancy is increasingly common, with millions of women sharing and seeking information on platforms like Instagram, Facebook, TikTok, YouTube, Snapchat, Pinterest, Twitter (X), and many others. These online forums can provide valuable benefits by offering social support, health education, and peer-to-peer advice. However, social media also carries risks: exposure to misinformation, unrealistic ideals, and negative social comparisons can increase anxiety, body dissatisfaction, and health confusion.

This review examines evidence from recent peer-reviewed studies (2019-2025) on the risks and benefits of social media use for pregnant women’s health. Using a comprehensive literature search, we identified relevant studies from the US, UK, Europe, and Asia. Key findings are that most pregnant women use social media for quick pregnancy information and emotional support, but the quality of information is uneven. Professional moderation and evidence-based content can enhance benefits, while unmoderated or highly visual content often leads to stress or misleading beliefs. Social media has the potential to positively supplement prenatal care if used carefully and with guidance, but women and healthcare providers should be aware of its pitfalls. Further research is needed on effective interventions to maximize benefits and minimize harms.

## Introduction and background

Social media platforms are now a widely used source of pregnancy information, education, and support. A survey of Australian women revealed that almost all used digital media, including apps and social platforms, during pregnancy due to ease of access [1]. In Turkey, all pregnant women in a 2023 sample used social media, with 71.6% showing signs of addiction linked to stress and anxiety, out of 358 pregnant women [2]. Similar findings were reported in Saudi Arabia, where many women rely on social media during pregnancy, though its psychological impacts vary [3]. Across global settings, pregnant women turn to digital sources for rapid answers and peer insights - often before consulting healthcare providers. Based on a detailed study, social media can be an effective tool for improving maternal health outcomes in Jordan, provided that healthcare organizations, lawmakers, and online platforms collaborate closely [4].

This review aims to summarize findings from over 20 peer-reviewed medical articles (out of 70 articles) published between 2019 and 2025, focusing on social media use during pregnancy and its implications for maternal and child health. I also bring 15 years of experience in obstetrics and gynecology, providing patient care in clinics across various countries, including Pakistan, the UAE, the UK, and Europe. We examine both the benefits (e.g., education, support) and risks (e.g., misinformation, stress) of digital engagement by pregnant women.

We conducted a structured literature review using PubMed, Scopus, ScienceDirect, and web searches to identify peer-reviewed articles on social media use during pregnancy. Our inclusion criteria focused primarily on studies published between 2019 and 2025, that were peer-reviewed, and based in the US, Europe, or Asia. We used keywords such as “social media,” “pregnancy,” “maternal health,” “digital health,” and “perinatal care.”

The search yielded over 70 articles, and after screening for relevance and study quality, 20+ unique publications were selected. These included qualitative interviews, surveys, and content analyses related to pregnant women’s engagement with social media. Studies were reviewed to extract data on sample characteristics, methodology, and key outcomes [1-20]. All data in this review are drawn from existing publications published recently, and no new data collection was performed. In addition, more than 15 years of clinical experience in obstetrics and gynaecology, gained in different countries while managing prenatal care for patients in a clinical setting, has been incorporated into this article.

## Review

Prevalence and patterns of use

Studies consistently show that pregnant women engage with social media for health information, reassurance, and community. In Australia, most women use digital media daily, turning to social platforms to ask pregnancy-related questions [1]. Similar trends were reported in Turkey, Ghana, and China, with digital engagement nearly universal among pregnant populations [2,5,6]. Women seek advice from other mothers, access peer stories, and follow influencers, often valuing social media over traditional health education [7].

Benefits of social media use

Social media offers emotional support, especially in high-risk pregnancies. In a study of Facebook support groups for women with complications, participants cited increased confidence, knowledge, and reduced feelings of isolation [8]. Similarly, midwife-led groups on Facebook improved trust and encouraged help-seeking [9]. These findings show that online communities, particularly those moderated by professionals, can effectively supplement traditional care.

Pregnant women use social media to learn about nutrition, birth planning, and baby care. A systematic review found that quality digital content can positively impact pregnancy preparation [10]. Moderated forums tend to provide accurate guidance, helping mothers build confidence [11]. Mobile health interventions that use social platforms have also improved outcomes like nutrition adherence and prenatal checkup compliance [12].

Many women feel empowered by online resources, particularly when access to health professionals is limited. In India, a WhatsApp-based group facilitated learning about gestational diabetes management. Similarly, pregnant women in Japan valued online antenatal classes during COVID-19 lockdowns, citing increased control over their health [13].

Risks of social media use

One of the most frequently cited risks is the spread of inaccurate or unverified health information. Studies show that a large proportion of maternal content on Instagram and Facebook lacks scientific backing [14]. Exposure to misinformation can delay help-seeking and encourage unsafe practices such as home births without supervision [15].

Social comparison on platforms like Instagram leads to increased body dissatisfaction and stress. In one study, women who viewed idealized pregnancy images reported lower self-esteem and increased worry [16]. Another found that excessive social media use contributed to sleep disturbances and mood issues [17].

Addiction to digital media during pregnancy is linked to depression, stress, and reduced physical activity. A Chinese study found that heavy app users had higher anxiety scores and poorer dietary habits [20]. Similar patterns were seen in Ethiopia and Qatar, where constant connectivity disrupted sleep and exercise routines [18,19].

Concerns also arise around data sharing. Pregnant women using social apps often share sensitive health data without understanding how it is stored or monetized [20]. Personalized ads may also exploit insecurities around pregnancy, feeding stress and anxiety.

To improve outcomes, professionals suggest greater integration of social media into prenatal care. Obstetricians should discuss patients’ digital behaviors and recommend reputable sources [21]. Training midwives to moderate forums and collaborate on app development can further ensure safety. Public health authorities can use social platforms for campaigns and myth-busting, especially in underserved areas.

The detailed study, “Browsing throughout pregnancy: the longitudinal course of social media use during pregnancy,” followed 1,135 pregnant individuals in the UK [22], measuring their social media use at 12, 20, and 28 weeks of gestation. Researchers found that time spent on social media, frequency of use, and signs of problematic use all significantly increased from early pregnancy to 20 weeks, then plateaued by 28 weeks. First-time mothers (primiparous) reported more time on social media at 20 weeks compared to those who had been pregnant before. These findings suggest that mid-pregnancy is a key period when social media use peaks, and healthcare providers may need to monitor and support healthy digital habits during this time to reduce potential mental health risks.

## Conclusions

Social media has emerged as a key resource for pregnant women worldwide, providing community support, access to information, and a sense of empowerment, particularly within moderated, evidence-based environments. Nonetheless, challenges such as misinformation, increased anxiety, and excessive use persist. Healthcare professionals have an important role in helping women make informed decisions about their digital engagement. When directed toward credible, medically verified sources - including authenticated social media channels and patient leaflets - social media can become a valuable tool for enhancing maternal and child health outcomes. Future policies and interventions should focus on maximizing these benefits while minimizing potential risks.
